# A Method of DDoS Attack Detection and Mitigation for the Comprehensive Coordinated Protection of SDN Controllers

**DOI:** 10.3390/e25081210

**Published:** 2023-08-14

**Authors:** Jin Wang, Liping Wang, Ruiqing Wang

**Affiliations:** 1 College of Computer Science and Technology, Zhejiang University of Technology, Hangzhou 310023, China; 2 School of Mathematics, Zhengzhou University of Aeronautics, Zhengzhou 450046, China

**Keywords:** software defined networking (SDN), distributed denial of service (DDoS), detection, mitigation

## Abstract

Software defined networking (SDN) improves the flexibility and programmability of the network by separating the control plane and the data plane and effectively realizes the global control of the network infrastructure. However, the centralized structure design of SDN exposes the controller to potential threats. Attackers have used the active flow table delivery mode to launch distributed denial of service (DDoS) attacks on the SDN controller, resulting in the controller failure and seriously affecting the network performance. To overcome this problem, this paper proposes a defense framework called CC-Guard. The framework consists of four modules: attack detection triggering, switch migration, anomaly detection, and mitigation. Among them, the attack detection trigger module improves the system’s timely response to DDoS attacks. The switch migration module effectively unclogs the controller congestion problem and provides convenience for network flow transmission. The anomaly detection module uses a coarse-grained method for two-stage detection, which improves the detection accuracy. The mitigation module uses the idea of cross-domain cooperation of the controller to clear the abnormal flow in the blacklist. Experimental results show that our proposed CC-Guard has real-time DDoS attack defense capability and high detection accuracy, as well as efficient network resource utilization.

## 1. Introduction

In recent years, with the rapid development of internet technology, the distributed control and management mode of the traditional network makes it difficult to upgrade to new network functions, resulting in the difficult implementation of network innovation [1]. As a new type of network architecture, SDN [2] decouples the traditional network architecture into an application plane, control plane, and data plane and adopts the centralized management mode of the control layer to make network control more flexible and centralized. Due to its openness and programmability, SDN has been widely used in network virtualization, cloud data center networks, wireless local area networks, and cloud computing [3].

The flexible network architecture of SDN provides convenience for network management, but its security problems have become increasingly prominent. OpenFlow [4] is a southbound interface protocol in SDN that defines the communication rules between SDN switches and SDN controllers. In the OpenFlow protocol, if the data packets cannot match the flow rules in the switch flow table, we call these packets table-miss. The OpenFlow protocol encapsulates the table-miss packet header into a packet_in message and sends it to the controller, and the controller provides the relevant forwarding strategy for it. This mode of operation greatly increases the possibility of the controller being subjected to flooding DDoS attacks. Attackers use the vulnerability of this working mode to create a large number of fake IP packets so that these fake packets cannot find the matching flow rules in the flow table in the switch, which causes the switch to generate a large number of packet_in messages and send them to the controller, resulting in the rapid depletion of CPU and storage resources of the controller, making the whole SDN network collapse.

At present, there are many DDoS attack detection and defense methods for SDN [5,6,7]. However, there are still shortcomings in the research on DDoS defense for SDN controllers. First of all, most existing detection methods are periodic. However, it is difficult to select an appropriate detection period. If the selected period is too long, the response time (from the initiation of the attack to the start of the attack detection) will be long, which makes the controller have to deal with a large number of attack packets or even destroys the controller. Contrarily, if the period is too short, attack detection will occur frequently, which will make the controller waste a lot of resources (such as CPU and memory) and affect the work efficiency of the controller. Therefore, the question of when to trigger the detection device needs to be solved urgently. Secondly, DDoS attacks will produce a large number of packet_in messages in a short time, which will quickly occupy the controller resources and affect the normal work of the detection equipment. Therefore, the task of unclogging the packet_in messages in the mitigation controller in time must be solved. Thirdly, with the gradual improvement in attack methods, DDoS attack flow is very similar to normal burst flow (flash events [8]), which makes DDoS attack detection more and more difficult. To improve the detection rate and real-time performance of abnormal flow detection, it is necessary to design an efficient abnormal flow detection model. Fourth, traditional methods for DDoS attack detection and mitigation consider a single controller, and these methods cannot be directly applied to a multi-controller scenario. In the multi-domain controller scenario, it is difficult to implement effective defense measures against large-scale distributed DoS attacks, so the defense measures in the multi-controller scenario need to be developed urgently.

In this paper, we propose a multi-domain comprehensive cooperative guard framework called CC-Guard to defend against DDoS attacks at the control layer. CC-Guard is divided into four modules: the attack detection trigger module, the switch migration module, the anomaly detection module, and the mitigation module at the control layer. When the ratio of the number of packet_in messages in the SDN network to the processing capacity of the controller is greater than a certain threshold, the system starts the switch migration module. The system selects the best target controller from the neighborhood and migrates some switches in the overloaded control domain to the target control domain to avoid controller overload in the attacked domain. Then, the anomaly detection module uses a two-stage detection method to detect the DDoS attack flow. In the first stage, it determines whether there was a DDoS attack based on the packet_in message characteristics. If the anomaly is detected, the DDoS attack flow is judged by the second stage of the deep learning method based on flow characteristics. Finally, the mitigation module deploys the abnormal flow blacklist in the controller and informs the neighborhood controller cross-domain through the east–west interface of the SDN controller, and then the attack host is isolated by issuing the flow rule command to prevent the attack from happening again. The following are our contributions with this paper.

- This paper proposes a multi-domain controller cooperation defense framework, CC-Guard, against DDoS attacks on SDN controllers. The mechanism includes the attack detection trigger module, the switch migration module, the anomaly detection module, and the mitigation module.
- In this paper, the DDoS attack detection trigger module is proposed to overcome the limitation of the fixed period detection method and realize adaptive fast detection.
- To prevent controller failure and impacts to the the operation of the detection module, this paper proposes the switch migration module for the first time to provide enough CPU computing resources for the subsequent start-up of the detection module.
- To improve the DDoS attack detection accuracy and reduce the false positive rate, this paper proposes a two-stage attack detection method. In the first stage, the entropy value of the source IP and destination IP is used for the coarse-grained judgment of whether the DDoS attack exists. If it exists, the CNN-GRU-Attention deep learning model is used for fine-grained detection of the DDoS flow. However, as DDoS attacks become more intelligent, the entropy method is not foolproof.
- To effectively mitigate DDoS attacks, this paper proposes a DDoS mitigation mechanism for attack blocking and flow cleaning. The SDN characteristics (query network topology and information of each switch) were used to identify the attack path and source of the attack flow. Finally, the OpenFlow protocol is used to send the flow table to the attack edge switch to clear the attack flow.

The rest of this paper is arranged as follows. Section 2 presents the related works. Section 3 presents the security challenges of DDoS attacks against SDN controllers. Section 4 describes the detailed design of the CC-Guard system. Section 5 presents the experiment and the experimental evaluation. Finally, Section 6 describes the conclusions and future work.

## 2. Related Works

SDN architectures are becoming a new network architecture of interest in academia and industry because of the manageability, flexibility, and programmability they provide to network infrastructures, enabling operations and maintenance staff to efficiently perform network monitoring, resource allocation, and dynamic provisioning. At the same time, the SDN architecture introduces many new types of security threats to the network. In particular, the problem of controller failure due to SDN-based DDoS attacks is of great concern to researchers. Researchers have proposed many solutions to mitigate DDoS attacks, mainly in terms of attack detection and SDN defense architecture design.

To date, a large number of SDN-based DDoS attack detection methods have been proposed, mainly based on the analysis of network flows. Nam et al. [9] combined statistical methods with neural network techniques to identify abnormal network behaviors. They used entropy to select specific features and then used a self-organizing map network (SOM) to classify network behavior. Deepa et al. [10] proposed an integrated technique for detection of SDN network flow anomalies. To improve the effectiveness of detection, they used nearest neighbor (KNN), Bayesian (NB), support vector machine (SVM), and self-organizing map network (SOM) to detect the abnormal behavior of data traffic in the SDN controller. Experimental results show that compared with the single learning algorithm, the ensemble method has a better detection rate, accuracy rate, and false alarm rate in machine learning. Li et al. [11] proposed a DDoS attack detection method based on deep learning, deep convolutional neural networks (DCNN) and deep stack autoencoder (DSAE). When constructing the deep learning model, in addition to 21 different types of fields extracted from the SDN data plane, five statistical features that can distinguish abnormal flow were designed at the same time. Experimental results show that the proposed method has high accuracy and is superior to traditional machine learning methods. Cui et al. [12] proposed an SDN-Anti defense mechanism to prevent DDoS attacks in SDNs. The proposed framework consists of four modules: attack detection triggering, attack detection, attack traceback, and attack mitigation modules. SD-Anti introduces the attack detection trigger module, which can quickly respond to DDoS attack detection and reduce the workload of controllers and switches. Abnormal flow is identified and judged through the attack detection module and attack traceability module, and finally, the mitigation module is triggered to effectively contain the attack traffic. Yang et al. [13] proposed a cross-platform collaborative attack detection framework for DDoS attacks, that is, coarse-grained detection in the data plane and fine-grained detection in the control plane. The proposed framework can greatly improve detection efficiency and reduce the delay and southbound channel overhead. Cui et al. [14] extracted the temporal behavior features of attacks and used the features to train a back propagation neural network (BPNN) model, which was then used to identify attacks. When a DDoS attack is detected, the defense module is activated, thus preventing the port from allowing entry of the malicious flow, and once the normal flow is detected, the port will automatically return to its normal state. Zhang et al. [15] proposed a two-stage detection method for DDoS attacks on SDN controllers. In the first stage, the information entropy detection method is used for coarse-grained detection of suspicious components and ports. In the second stage, a stacked sparse autoencoder (SSAE) and support vector machine (SVM) hybrid model is used to perform fine-grained detection of data packets. Finally, the controller executes a defense strategy to intercept the attack. Experimental results show that the two-stage detection mechanism has higher detection accuracy and efficiency in the DDoS attack scenario, and has obvious advantages.

SDN-based DDoS attack defense schemes are considered from the overall architecture of SDN, such as adding additional devices in the data plane and control plane or using nearby idle network devices in the domain to alleviate the impact of SDN DDoS attacks so that the network can work normally. Shin et al. [16] proposed a method to mitigate DDoS attack saturation by extending the existing data plane. They added two modules to AvantGuard: the connection migration module and the execution trigger module. The connection migration module can transfer the failed TCP sessions in the data, which greatly reduces the amount of interaction between the data plane and the control plane during the attack. Before notifying the controller, the execution trigger module collects the network status information and packet payload information and activates some flow rules according to the situation so that the control plane can speed up the timely response and detection of the ever-changing flow in the data plane. Experiments show that AvantGuard is more scalable and responsive to dynamic network threats. Wang et al. [17] proposed a lightweight, efficient, and protocol-independent security defense framework called FloodGuard to overcome the shortcomings of AvantGuard. The framework consists of two modules: the active flow rule installation module and the packet migration module. The active flow rule installation module can actively obtain active flow rules by viewing the runtime status of the SDN controller, which will help to protect the execution of network policies. The packet migration module temporarily caches the failed packets in the newly added switch devices and submits these packets to the controller at a certain rate using round-robin scheduling to prevent controller resources from being exhausted. However, Gao et al. [18] proposed an efficient and protocol-independent SDN defense framework to overcome the shortcomings of AvantGuard and FloodGuard. It is located between the control plane and the data plane and conforms to the OpenFlow protocol without additional equipment. The framework consists of two modules: the detection module and the mitigation module. The detection module uses the new characteristic frequency to accurately identify DDoS attacks. The mitigation module uses three new techniques to effectively mitigate the attack flow: table-miss engineering to prevent bandwidth depletion, packet filtering engineering filters attack flows and saves controller resources, and flow rule management project eliminates most of the useless flow table entries in the switch flow table. The framework can accurately identify and effectively mitigate SDN DDoS attacks. In addition, some researchers have introduced additional controllers to mitigate the impact of DDoS attacks on SDNs. Macedo et al. [19] proposed PATMOS, a method to mitigate SDN DDoS attacks through controller clustering, which is mainly divided into three steps: (1) Identify overloaded controllers. (2) Choose the best controller to coordinate mitigation. (3) Operate the controller to minimize the impact of DDoS attacks. Similarly to this work, Wang et al. [20] proposed a solution to protect against control plane DDoS attacks, namely SafeGuard. The scheme includes two modules: the anomaly detection module and the dynamic defense module. The anomaly detection module is deployed in the switch to detect abnormal flow. The dynamic defense module finds the best proxy controller and maps the unmatched flow of the victim switch onto the best proxy controller. Then, the agent controller issues control messages to the switch, discards abnormal packets, and cleans the corresponding flow entries in the switch to release the space occupied by the attack flow. The proposed scheme has high resource utilization and can effectively alleviate the impact of DDoS attacks on controller resource consumption. Wu et al. [21] proposed a new mitigation scheme, FlowMitigation, in order to resist DDoS attacks in SDNs. The proposed scheme uses a slave controller as an alternative to tolerating flooding requests from the master controller. Then, these requests are re-forwarded to the main controller by using the round-robin scheduling method. In order to prevent the resource depletion of the main controller, an adaptive rate adjustment method is proposed to dynamically adjust the forwarding rate. Experimental results show that the proposed method can effectively mitigate DDoS attacks and has better performance in terms of request response time, packet loss rate, and mitigation time.

To sum up, the methods to prevent DDoS attacks against SDN controllers still have the following shortcomings. First of all, most detection and defense methods only consider how to improve the detection efficiency, without considering the time delay when the controller starts the detection module, which will cause a large number of abnormal flow packets to congest the controller before the controller detects them, affecting the normal work of the controller. Secondly, most DDoS attack mitigation methods based on SDN controllers only consider the distinction between normal and abnormal flow, without considering the situation of sudden normal flow, which can easily lead to a high false detection rate. Finally, most works in the literature only consider how to detect DDoS attacks and do not consider how to effectively alleviate abnormal flow. To solve the above problems, this paper proposes the CC-Guard defense framework from the perspective of multi-domain controller cooperation, which can not only ensure that the controller has enough computing resources to drive the detection module, but also reduce the false positive rate of DDoS attacks and launch effective mitigation measures against DDoS attacks.

## 3. Security Challenges

**Figure 1:**
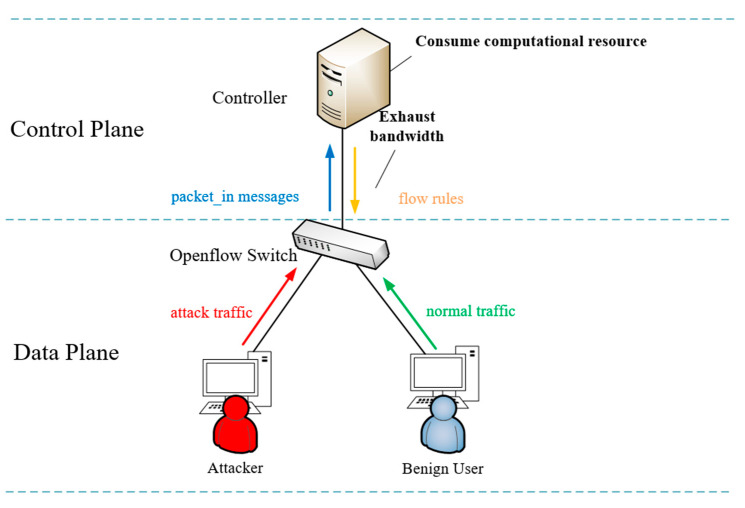
Packet_in flooding attack.

The DDoS attacks in SDN are different from traditional network attacks. In OpenFlow networks, attackers can exploit the communication overhead between the data layer and the control layer to launch attacks. Attackers use malicious software to invade one or more network hosts and launch TCP/UDP flood attacks on the attack targets through the invaded hosts to generate a large number of fake data packets (called table_miss packets) that falsify fields in the OpenFlow network in a short time. However, these table_miss packets fail to match the flow rules in the switch, which triggers the switch cache to send a large number of packet_in messages with table_miss packet header information to the controller. To make things worse, the OpenFlow specification v1.4 [4] requires that the entire table_miss packet be included in the packet_in message when the memory of the switch is full. Finally, a large number of packet_in messages cause serious resource depletion for the controller, the bandwidth between the controller and the switch, and the switch, so that the controller can not work normally. The packet_in flooding attack scenario is shown in Figure 1, where malicious hosts continuously inject attack streams into switches, causing the entire network to crash.

**Figure 2:**
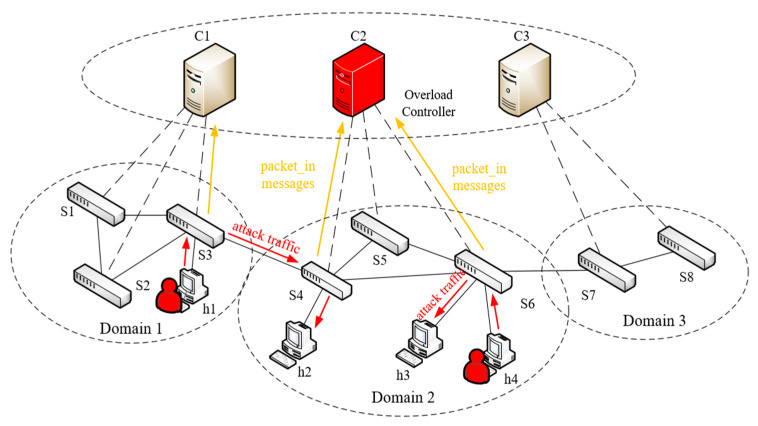
Controller targeted by DDoS attack in SDN.

In multiple control domain scenarios, to achieve DDoS attacks on SDN controllers more covertly, attackers usually launch DDoS attacks from the neighboring domain of the target controller, which greatly increases the difficulty of detection and mitigation. As shown in Figure 2 (this paper assumes that there is only one controller in each control domain), host 1 launches an attack on host 2, causing switches S3 and S4 to generate many packet_in messages and send them to controllers C1 and C2. Host 4 launches an attack on host 3, causing switch S6 to generate many packet_in messages and send them to controller C2, which eventually leads to the overload of controller C2 and affects its normal operation.

At present, many research methods have been devised to address DDoS attacks in a single control domain, but these methods cannot be effectively applied to DDoS attacks in cross-domain scenarios. However, there is no complete scheme to prevent DDoS attacks between multiple control domains. Therefore, this paper considers DDoS attack defense methods in multiple control domains by using threat information between north and south interfaces and network resources between east and west interfaces. From the perspective of the north–south interface, the threat situation of DDoS attack flow in the data plane cannot be completely reported to a single controller to avoid controller failure. Considering the east–west interface, network threat information within multiple control domains should be shared to quickly identify attack scenarios, track attackers more accurately, and prevent DDoS flow from spreading among multiple control domains.

## 4. System Design

To effectively prevent controller DDoS attacks, we propose the CC-Guard defense framework, which is deployed on the SDN controller and consists of four modules. It mainly includes the attack detection trigger module, the switch migration module, the anomaly detection module, and the mitigation module. The detailed description of each module is as follows. The key parameters in each module are shown in Table 1.

### 4.1. Attack Detection Trigger Module

At present, many DDoS attack detection methods have been proposed in SDN, mainly focusing on detection and defense mechanisms [22,23]. Most of the existing detection methods are usually scheduled by periodic triggers. But, it is very difficult to choose an appropriate detection period. If the chosen period is too large, the response time will be too long, which forces the controller and switch to deal with a large number of attack packets and may cause the controller to crash. In contrast, if the period is too small, the detection module will be started more frequently, which leads to a waste of controller resources (i.e., CPU and network bandwidth) and affects controller efficiency. Therefore, as an important factor affecting detection efficiency and system performance, the trigger mechanism of attack detection is very worthy of scholars’ attention.

When a DDoS attack occurs, the number of packet_in messages will increase sharply, resulting in a decline in controller processing capacity and the depletion of controller resources. Therefore, we consider the ratio of the number of packet_in messages per unit time t to the processing capacity of the controller as the prerequisite for triggering the CC-Guard system.

(1) $$\delta_{j}=\frac{\sum_{i=1}^{M}\lambda_{i}}{\eta_{j}}$$

In Equation (1), M represents the number of switches managed in controller $C_{j}$, $\lambda_{i}$ represents the flow request rate of switch $S_{i}$, $\eta_{j}$ represents the processing capacity of the controller $C_{j}$, and $\delta_{j}$ represents the threshold for determining whether to start the system detection module. If $0.8\leq \delta_{j}\leq 1$, it indicates that controller $C_{j}$ is about to be overloaded, at which point the system trigger mechanism is started.

### 4.2. Switch Migration Module

When the network is started, the burst situation of normal flow is very similar to that of a high-speed DDoS attack. A large number of packet_in messages will quickly occupy the controller, leading to the exhaustion of controller computing resources and space resources, and even directly affecting the normal operation of the SDN. Therefore, it is necessary to dredge and relieve the SDN controller. In this section, we will look at how to migrate the switch to balance the load across multiple controllers.

The idea of the switch migration module is to migrate the switches in the overloaded controller to other controllers to alleviate the load on the attacked controller and ensure that each controller can work properly. The module is divided into three stages: (1) the overloaded controller identification stage; (2) the switch migration phase; and (3) the switch mapping phase. In the overloaded controller identification stage, the controller under load and the switch to be migrated are found based on the abnormal flow impact results. In the switch migration phase, the target controller is selected from the controller cluster and migrated to the target controller domain, thereby reducing the workload of overloaded controllers and enabling each controller to work normally. The switch mapping phase migrates the switch to be selected to the target controller.

#### 4.2.1. Identification of Overloaded Controller

When subjected to DDoS attacks, controllers are overloaded by processing malicious flow requests, so it is important to identify overloaded controllers under DDoS attacks. The reason for overload is that the number of packet_in messages sent by the switch is greater than the processing capacity of the controller, which leads to the load on the controller. Therefore, we identify the overloaded controller according to Equation (2). If the number of packet_in messages of controller $C_{j}$ is greater than 0.8 times the controller’s processing capacity, we consider that the controller is overloaded and switch migration is required.

(2) $$\sum_{i=1}^{M}\alpha_{i}\times f_{ij}>0.8\times \eta_{j}$$

In Equation (2), $\alpha_{i}$ represents the number of packet_in messages sent by switch $S_{i}$ to controller $C_{j}$; $\eta_{j}$ represents the processing capacity of controller $C_{j}$; $f_{ij}$ represents the connection between switch $S_{i}$ and controller $C_{j}$. If $f_{ij}$ is 1, it means that controller $C_{j}$ has a connection with switch $S_{i}$; otherwise, if $f_{ij}$ is 0, it indicates that the controller $C_{j}$ is not connected to switch $S_{i}$.

#### 4.2.2. Optimal Domain Migration Strategy for Switches

The essence of the switch migration problem is how to migrate switches in the overloaded domain to other controllers so that the load on each controller is balanced. The main algorithm process is as follows: Firstly, the candidate set $S_{M}$ of migration switches is found from the overloaded controller domain according to Equation (2). Then, we select the target controller from the normal controller and set $C_{N}$ according to the controller processing capacity threshold. The selection of the target controller is calculated with the objective of minimizing the switch migration time delay (time delay refers to the sum of the shortest physical distances of the switch $S_{i}$ to be migrated from the overloaded controller $C_{j}$ to the target controller $C_{j^{\prime }}$, as shown in Equation (3). This problem is an objective optimization problem and can be solved using the genetic algorithm [24]. Finally, the controller with the minimum function value is selected as the target controller, and the selected switch is migrated to the target controller. The pseudo-code of the switch migration algorithm is shown in Algorithm 1.

(3) $$\min E=\sum_{i=1}^{M}m_{i}\left(H_{ij}+H_{ij^{\prime }}\right)$$

(4) $$s.t.\;\;\;\;\sum_{i=1}^{M}\alpha_{i}\times f_{ij^{\prime }}^{\prime }<0.8\times \eta_{j^{\prime }}$$

In Equation (3), $H_{ij}$ represents the shortest distance between the switch $S_{i}$ to be migrated and the overloaded controller $C_{j}$; $H_{ij^{\prime }}$ represents the shortest distance between the switch $S_{i}$ to be migrated and the target controller $C_{j^{\prime }}$ (distance refers to the physical distance in the network topology). The state of the switch and controller is mapped from $F=(f_{ij}{)}_{M\times N}$ to $F=(f_{ij^{\prime }}^{\prime }{)}_{M\times N}$, and the value of $m_{i}$ is calculated according to Equation (5). If the value of $m_{i}$ is 0, it means that switch $S_{i}$ has not changed state. If the value of $m_{i}$ is 1, it means that switch $S_{i}$ has a state change.

(5) $$m_{i}=\left\{\begin{array}{l} 0,f_{ij}=f_{ij^{\prime }}^{\prime }=1\;\&\;j=j^{\prime }\\ 1,f_{ij}=f_{ij^{\prime }}^{\prime }=1\;\&\;j\neq j^{\prime } \end{array}\right.$$

**Algorithm 1:** Target controller selection algorithm**Input:** $\mathrm{controller}\;\mathrm{set}\;C_{n}$$,\;\mathrm{switch}\;\mathrm{set}\;S_{m}$; **Output:** $\mathrm{target}\;\mathrm{controller}\;C_{t}$$,\;\mathrm{migration}\;\mathrm{switch}\;S_{t}$;(1) **for**
$C_{i}\in C_{n}$ **do**(2) **if**
$\sum_{i=1}^{M}\alpha_{i}\times f_{ij}>0.8\times \eta_{j}$
(3) **then**
$C_{over}\leftarrow C_{i}$
(4) **end if**
(5) **end for**
(6) $\mathrm{Get}\;\mathrm{normal}\;\mathrm{controller}\;\mathrm{set}\;C_{nor}=C_{n}-C_{over}$
(7) $\mathrm{Select}\;\mathrm{switch}\;\mathrm{set}\;{S_{m}}^{\prime }$
$\;\mathrm{from}\;\mathrm{controller}\;C_{\mathrm{over}}$
(8) **While**
$C_{i}\in C_{\mathrm{nor}}\;\&\;S_{i}\in {S_{m}}^{\prime }$ **do**(9) The GA algorithm is used to calculate minE$\;\&\;\sum_{i=1}^{M}a_{i}\times f_{ij^{\prime }}^{\prime }<0.8\times \eta_{j^{\prime }}$
(10) **end while**


#### 4.2.3. The Switch Mapping Phase

From the above description of the switch migration phase, we know the switch and the target controller to be selected for migration. This phase tells us how to migrate switch $S_{t}$ from overloaded controller $C_{\mathrm{over}}$ to target controller $C_{t}$. The switch $S_{t}$ sends a Remap message to the destination controller $C_{t}$, which replies with a Remap-Begin message after receiving the remap message and selects a random number to start the countdown. If the switch mapping is completed before the countdown to 0 s, $C_{t}$ sends an access control message to the switch $S_{t}$ to be migrated and broadcasts the update message to the entire network. However, if the countdown times out, the countdown is reset, and the switch mapping process starts again.

### 4.3. Cross-Domain Routing

The process of cross-domain routing for data packets is as follows. Firstly, each domain controller periodically obtains the network link information in the control domain through the LLDP protocol. Then, each controller interacts with the network link state information through the east–west interface to obtain the link reachability information. Finally, the switch requests the controller in its domain to issue flow rules and forward the packets. An example is given as follows.

**Figure 3:**
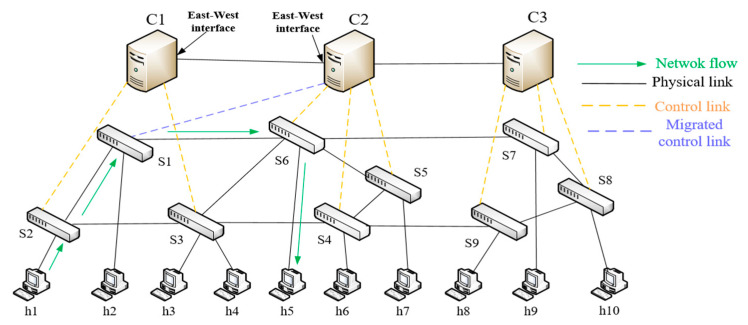
Cross-domain routing graph.

In Figure 3, S1 is the migrated switch. After migration, switches S2 and S3 are managed by controller C1, and S1, S4, S5, and S6 are managed by controller C2. First, controllers C1 and C2 obtain the link information in their respective domains through the LLDP protocol. Then, C1 and C2 interact with their link information through the east-west interface. Assuming a new data packet flows from host h1 to host h5, links S2→S1 and S1→S6 have reachability, and the routing path of the data packet is h1→S2→S1→S6→h5. At this time, h1 sends a new data packet to S2, then S2 sends a packet_in message to controller C1, and controller C1 generates the corresponding flow rule after receiving the request message from S2 and sends it to S2 through the packet_out messages. The data packet is forwarded to S1 through this flow rule. Similarly, the data packet continues to be forwarded through S1 and S6 until the new data packet reaches host h5.

### 4.4. Anomaly Detection Module

**Figure 4:**
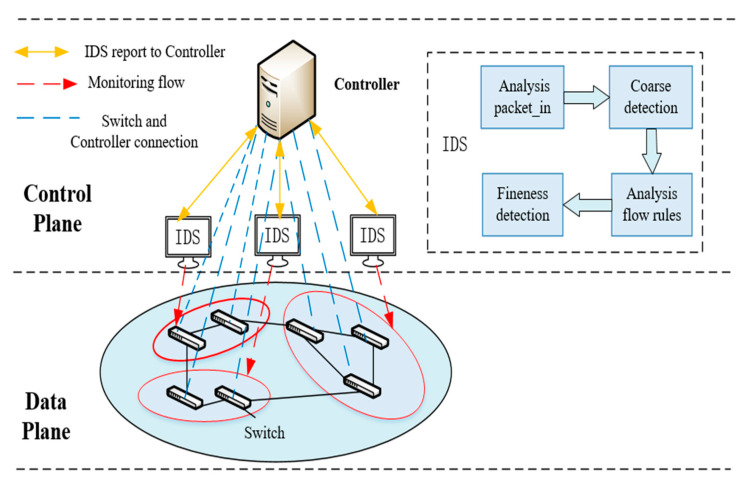
Schematic diagram of the anomaly detection module.

The anomaly detection module in this paper is deployed in the controller of the SDN network, as shown in Figure 4. Due to the large flow of distributed DDoS attacks, using a single abnormal flow detection device (IDS) easily causes overloads and increases detection delay. To overcome this problem, we use multiple detection devices to cooperate and detect in parallel. The deployment of the detection device is a single-objective optimization problem. The optimal value of the detection device load balancing degree can be obtained by the genetic algorithm. The objective function is as follows:

(6) $$LD_{j}=\sum_{i=0}^{M}\alpha_{i}\times g_{ij}$$

(7) $$\bar{LD}=\sum_{j=1}^{K}LD_{j}/K$$

(8) $$\min\varepsilon =\sqrt{\sum_{j=1}^{K}{\left(LD_{j}-\bar{LD}\right)}^{2}/K}$$

(9) $$s.t.\;\;\sum_{i=1}^{M}g_{ij}=1,\forall i$$

In Equation (6), $\alpha_{i}$ represents the number of packet_in messages sent by switch $S_{i}$ to intrusion detector $IDS_{j}$; $g_{ij}$ represents the connection relationship between switch $S_{i}$ and $IDS_{j}$, and $LD_{j}$ represents the load of $IDS_{j}$. In Equation (7), $\bar{LD}$ represents the IDS cluster average load. The objective function ε is shown in Equation (8), which represents the load balancing degree of IDS (that is, the situation where all IDS in the control plane have load differences), and the smaller ε means the load balancing of IDS. The mapping relationship between switches and IDS are constructed as a 0–1 matrix to represent $G={\left(g_{ij}\right)}_{M\times K}$, M represents the number of switches, K represents the number of IDS, and if the value of $g_{ij}$ is 1, it means that switch $S_{i}$ is connected to $IDS_{j}$, otherwise, the value of $g_{ij}$ is 0. Equation (9) indicates that each switch belongs to only one IDS.

Next, the anomaly detection module performs anomaly detection on flow data, a process that mainly includes two stages. In the first stage, by collecting the packet_in messages in the SDN controller, the entropy value of data features in a fixed window is calculated and compared with the set threshold. Once the detected entropy value exceeds the normal threshold, the flow is suspected to be an attack flow. In the second stage, the CNN-GRU-Attention deep hybrid model is used to further detect the flow characteristics to confirm whether DDoS attacks have occurred in the network. The pseudocode of the two-stage anomaly detection algorithm is shown in Algorithm 2. The detailed steps of each stage are described below.
**Algorithm 2:** Two-Stage detection algorithm**Input:**$\;\mathrm{packet}\_\mathrm{in}\;\mathrm{messages},\;\mathrm{flow}\;\mathrm{data},\;\mathrm{thresholds}\;\delta_{1}$$,\;\delta_{2}$, CNN-GRU model parameter values**Output:** detection results(1) Collect packet_in messages(2) $\mathrm{Computing}\;E_{IPS}$$,\;E_{IPD}$
(3) **if**
$E_{IPS}<\delta_{1}\;\&\;E_{IPD}<\delta_{2}$ then(4) **then** the flow is a normal burst flow(flash flow), generating a flow rule and forwarding it.(5) **else**
(6) **then** the flow is a suspect flow, skip to step 8(7) **end if**
(8) Collect flow data features(9) Results = CNN-GRU-Attention (flow data)(10) Output Results

#### 4.4.1. The First Stage Detection Method Based on Entropy

Both high-speed DDoS attacks and flash events will generate a large number of data packets in a short time, and these data packets cannot match the flow rules in the switch, so a large number of packet_in messages will be generated and sent to the controller, causing the controller service interruption. Therefore, the traffic characteristics of flash events are similar to high-speed DDoS attack flows, so it is difficult to distinguish them from DDoS flows. So, in the early stage of detection, we use the method of information entropy to preliminarily judge the abnormal flow and exclude the false detection caused by flash events.

Based on the above discussion, we propose to calculate the entropy of the source IP and destination IP in the packet_in message to determine whether an anomaly occurs. The entropy value calculation is shown in Equation (10), and the source IP entropy and destination IP entropy calculations are shown in Equations (11) and (12), respectively. If the above two indicators do not satisfy $E_{IPS}<\delta_{1}\;\&\;E_{IPD}<\delta_{2}$, the preliminary detection will judge that there is a suspicious flow; otherwise, the judgment will be for a flash flow.

(10) $$EntropyVal=-\sum_{i=1}^{N}P_{i}\log_{2}P_{i}$$

In Equation (10), $P_{i}=n_{i}/S$ is the probability of samples i per unit of time. The data sample $x=\left\{n_{i};i=1,2,3,\cdots ,N\right\}$ indicates that the sample i occurred $n_{i}$ times per unit time t. $S=\sum_{i}^{N}n_{i}$ denotes the total number of samples per unit time t. When the sample values are most concentrated, the entropy is 0, and all of the sample values are the same. Entropy is highest when the distribution of samples is most spread out, at which point all sample values are different. This method does not need to train the model and has low computational complexity and a short detection time.

1. Entropy of the source IP

When a DDoS attack occurs, attackers usually randomly generate many false source IP packets, causing an increase in the source IP address and an increase in the source IP entropy. When a flash event occurs, thousands of legitimate users accessing a specific host or server can also cause the source IP entropy to increase.

(11) $$E_{IPS}=-\sum_{i}^{n}P_{IPS_{i}}\log_{2}P_{IPS_{i}}$$

In Equation (11), $P_{IPS_{i}}$ denotes the frequency of occurrence of the source IP entropy value in packet_in messages per unit time t.

2. Entropy of the destination IP

When a DDoS attack occurs, as the attack target is an SDN controller, the attacker can widely send the attack stream to the target host, resulting in a high entropy value for the destination IP of the attack stream. However, the characteristic of flash events is that different source IP addresses flow into a small number of target hosts, resulting in a smaller entropy value for the destination IP. Therefore, the IP entropy serves as one of the important criteria for distinguishing abnormal flows.

(12) $$E_{IPD}=-\sum_{i}^{n}P_{IPD_{i}}\log_{2}P_{IPD_{i}}$$

In Equation (12), $P_{IPD_{i}}$ denotes the frequency of occurrence of the destination IP entropy value in packet_in messages per unit time t.

To effectively distinguish abnormal flow, the threshold setting is important. The setting of the threshold follows the hypothesis testing method in statistics. Confidence intervals for entropy are calculated from the number of samples *N*, the mean value *µ* of the samples, and the standard deviation σ of the samples. Then, the detection threshold is determined based on the average of the confidence intervals. The confidence interval $\left[a,b\right]$ is calculated as shown in Equation (13).

(13) $$[a,b]=\left[\mu -\frac{Z_{1-\alpha /2}}{\sqrt{N}}\sigma ,\mu +\frac{Z_{1-\alpha /2}}{\sqrt{N}}\sigma \right]$$

In Equation (13), a stands for the confidence level, which is a predetermined standard used in hypothesis testing to evaluate events with low likelihood. According to the standard normal distribution table, when the α is 5%, the $Z_{1-\alpha /2}$ is 1.96 [25].

#### 4.4.2. The Second-Stage Detection Method Based on CNN-GRU-Attention

Through the first stage of coarse-grained detection, it was preliminarily determined whether there were suspicious flows in the SDN network. In order to improve the detection accuracy, the deep learning hybrid model CNN-GRU-Attention is used for fine-grained detection in the second stage. The specific process is described as follows.

1. Feature selection

In machine learning, feature selection directly affects the performance of the overall algorithm. Selecting good features will improve the accuracy of the algorithm, but selecting too many irrelevant features will increase the complexity of the algorithm and affect its accuracy. To better validate the superiority of the proposed anomaly detection model, we selected 19 commonly used fields from the switch flow table as feature inputs. Among them, there were 12 matching domain fields (i.e., fields in the packet header), two counter fields, two timeout fields, one priority field, one action field, and one cookie field in the flow table entries that could reflect the characteristics of DDoS attack flows. In addition, according to the nature of the flow features, we manually constructed five statistical features as another part of the feature input to improve the accuracy of the CNN-GRU-Attention anomaly detection model. The statistical features are described as follows. The input features of the CNN-GRU-Attention model are shown in Table 2.

(1) Average value of packets in the flow rule (Avg_packets)

DDoS attacks typically forge a large number of source IP address packets to attack the target to prevent the defender from tracking the source. Therefore, the number of packets under each flow rule in the flow table will decrease sharply. Conversely, using a fixed source IP address for DDoS attacks can significantly increase the number of packets under each flow rule.

(14) $$avg\_packets=\frac{n\_packets}{duration}$$

In Equation (14), avg_packets denotes the average of the number of packets per unit time t in flow rule i, and duration denotes the duration of flow rule i.

(2) Average of packet sizes in the flow rule (Avg_bytes)

DDoS attacks usually send a large number of packets in a short period of time, but the size of the packets is much smaller than normal packets, so the size of the packets can be one of the important criteria to distinguish whether an attack has occurred or not.

(15) $$avg\_bytes=\frac{n\_bytes}{duration}$$

In Equation (15), avg_bytes denotes the average value of packet size per unit time t in flow rule i, and duration denotes the duration of flow rule i.

(3) Average survival time of the flow rule (Avg_duration)

DDoS attacks usually send a large number of fake IP packets in a short period of time, with the aim of taking up the target’s resources. The duration of these fake packets surviving under the flow rules is short compared to normal packets. Conversely, with fixed source IP addresses for DDoS attacks, the flow rule survival time increases significantly. Flow rule survival time can, therefore, be used as one of the important criteria to distinguish whether an attack has occurred or not.

(16) $$avg\_duration=\frac{\sum_{i=1}^{n}duration_{i}}{n}$$

In Equation (16), avg_duration denotes the average duration of each flow rule, $duration_{i}$ denotes the duration of flow rule i, and n is the number of samples.

(4) Survival degree of the flow rule (survival_degree)

Similarly, flow rules generated under DDoS attacks are less survivable than normal flow rules.

(17) $$survival\_degree=\frac{duration_{i}}{idle\_time_{i}}$$

In Equation (17), $duration_{i}$ is the duration of flow rule i, and $idle\_time_{i}$ is the idle time of flow rule i.

(5) Upper and lower flow ratio (Ratio_packets)

When a DDoS attack occurs, a huge amount of traffic flows to the victim, and the victim cannot respond properly. As a result, the ratio of upstream to downstream flow during DDoS is abnormal. Here, we define the packets from the switch to the terminal as the uplink flow and the packets from the terminal to the switch as the downlink flow. In the case of a DDoS attack, the ratio will be less than 1.

(18) $$ratio\_packets=\frac{pfd_{i}}{pbk_{i}}$$

In Equation (18), $pfd_{i}$ denotes the packets sent from the switch viewpoint per unit time t, and $pbk_{i}$ denotes the number of packets received from the switch viewpoint per unit time t.

2. CNN-GRU-Attention detection model

**Figure 5:**
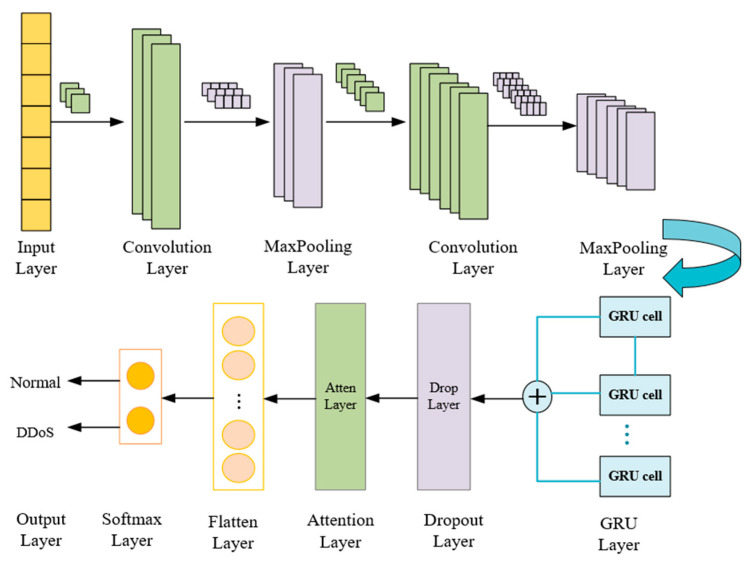
CNN-GRU-Attention structure graph.

Deep learning has achieved success in the field of intrusion detection research because of its powerful feature extraction ability. CNN [26] abstracts local features of data through convolution operations to realize data feature extraction in the spatial dimension. GRU preserves the historical information of data through the connection of neurons in the hidden layer and realizes the data features extracted in the time dimension. The self-attention mechanism [27] can capture relevant spatiotemporal features more accurately, to reduce the negative impact caused by error or redundant feature information and improve classification performance. In this paper, CNN and GRU are used to extract the spatial and temporal features of network flow, respectively, and the self-attention mechanism is introduced into the CNN-GRU model to improve the classification accuracy of the detection algorithm. The CNN-GRU-Attention model structure is shown in Figure 5, and the structural parameters are shown in Table 3.

Due to significant differences in the numerical values of the collected network flow data, to improve the accuracy of training, the sample data is normalized and then divided into the training set and test set according to 8:2. The sample labels are converted into binary (0 and 1) representation using one-hot encoding. In the CNN-GRU-Attention model structure, the convolution kernel size in the convolutional layer is 3, the step size is 1, and the RELU activation function is used to prevent the gradient from disappearing, increase the nonlinearity of the network, and make the network training faster. The pooling layer uses Maxpooling, and the kernel size is 2. The Maxpooling layer has the characteristics of translation invariance, which can reduce the parameters to reduce the dimension and prevent overfitting while retaining the main features. The activation function of the GRU layer uses the hyperbolic tangent function Tanh, and the output mapping is between [−1, 1], with a limited output range and stable optimization. Finally, the softmax function is used to classify the network flow.

A. CNN layer

The convolutional neural network, as a feedforward neural network, uses the idea of local correlation and weight sharing to reduce neural network parameters and achieve higher training efficiency. CNN is mainly composed of three parts: convolutional layer, pooling layer, and fully connected layer. The convolution layer extracts the feature information of the data by performing correlation operations on the matrix of each channel through the convolution kernel and increases the expression ability of the model through the non-activation function. The convolutional layer Equation is as follows:

(19) $$x_{i}^{l}=f\left(W_{i}^{l}*x^{\left(l-1\right)}+b_{i}^{l}\right)$$

In Equation (19), $x_{i}^{l}$ is the *i*th feature in the output of layer l; f is the network nonlinear function RELU; $W_{i}^{l}$ is the weight of the ith convolution kernel in the lth layer; ∗ denotes the convolution operation; $x^{\left(l-1\right)}$ is the input of layer l. $b_{i}^{l}$ is the bias of the kernel of the ith convolution kernel in the lth layer.

The role of the pooling layer is to reduce the size of the feature map and simplify the complexity of network calculation. At the same time, the features are compressed to retain salient features and prevent overfitting. Pooling layers are usually sandwiched between successive convolutional layers. The function of the fully connected layer is to integrate the features calculated by the convolutional layer and the pooling layer into the sample space. Fully connected layers are usually placed at the end of the convolutional neural network architecture.

B. GRU layer

**Figure 6:**
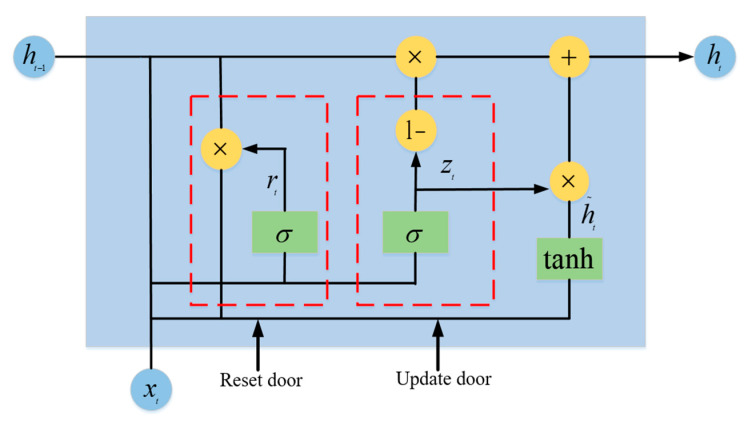
GRU unit structure.

After multiple convolutions and pooling to extract more accurate frequency and spatial domain features, the GRU layer is used as the input data of the GRU network. Among them, the update gate of GRU is close to 0, which means that some information about the hidden state of the previous layer is forgotten in this hidden layer. The update gate is close to 1, indicating that this hidden layer continues to be retained. When the reset gate of GRU is close to 0, it means that some information at the previous time is forgotten in the current memory content. The reset gate is close to 1, indicating continued retention in the current memory content. Then, the two pieces of information are added and passed through the tanh activation function to normalize the result to between [−1, 1]. Therefore, the memory content of this moment is composed of two parts: one is the reset gate to store the important information related to the past, and the other is the important information input at the current moment. These two parts compose all of the memory content of the current moment to extract the stream feature. The structural diagram of the GRU unit is shown in Figure 6.

Two gates are obtained by the last transmission state $h_{t-1}$ and the input $x_{t}$ of the current node, the update gate $z_{t}$ and reset gate $r_{t}$ are represented as follows:

(20) $$z_{t}=\sigma \left(W_{z}h_{t-1}+U_{z}x_{t}+b_{z}\right)$$

(21) $$r_{t}=\sigma \left(W_{r}h_{t-1}+U_{r}x_{t}+b_{r}\right)$$

(22) $${\tilde{h}}_{t}=\tanh\left(W_{h}x_{t}+U_{h}\left(r_{t}⊗h_{t-1}\right)+b_{h}\right)$$

(23) $$h_{t}=\left(1-z_{t}\right)⊗h_{t-1}+z_{t}⊗{\tilde{h}}_{t}$$

In Equations (20)–(23), $W_{z}$ and $b_{z}$ are the weight and bias of the update gate, respectively; $W_{r}$ and $b_{r}$ are the weight and bias of the reset gate, respectively; $\tilde{h_{t}}$ is the candidate state at time t; σ is the sigmoid function.

C. Self-attention layer

For each flow, not all bytes are equally important for classification. To make the relatively important information in the stream have a strong influence on the classification results, this paper introduces the attention mechanism. In the attention mechanism, the core operation is the calculation of the weight of each element. In this paper, the feature vector $h_{t}$ output by the feature extraction layer is used as the input of the attention layer, and the flow feature vector s is obtained by weighted summation of the hidden states at each time step.

(24) $$s=\sum_{t=1}^{N}a_{t}h_{t}$$

(25) $$a_{t}=\frac{\exp(e_{t})}{\sum_{t}\exp(e_{t})}$$

(26) $$e_{t}=\tanh(W_{h}h_{t}+b_{h})$$

In Equations (24)–(26), $a_{t}$ is the weight coefficient of $h_{t}$; $e_{t}$ is the hidden layer generated by $h_{t}$ through a feedforward neural network layer; $W_{h}$ and $b_{h}$ are the weight matrix and bias, respectively.

### 4.5. Mitigation Module

In the cross-domain scenario, an attacker can construct many attack packets using the forged IP addresses or MAC addresses, so that the switch generates packet_in messages from the attacking host to the target host. Finally, many packet_in messages are gathered in the controller, causing the controller to fail. As shown in Figure 2, the controller in the target domain is subjected to DDoS attacks in the local control domain and the neighbor domain. In order to effectively mitigate such DDoS attacks, this paper proposes a cross-domain cooperation defense method based on the advantage of SDN’s real-time control of the network. This method can permanently offline the attacking host from the network and effectively prevent the attacker from tampering with the IP address and then injecting attack flow into the network again. The specific defense process is as follows:

1. Extract the information. Once the attack flow is detected, the attack source IP, destination IP, switch ID, switch ingress port, and the timestamp when the switch sends packet_in messages to the controller are extracted from the flow table and put into the blacklist in the controller.
2. Locate the attack source. The flow data in the blacklist are grouped according to the source IP and destination IP fields, and then each group of flow data is sorted in descending order according to the timestamp field to find the smallest timestamp flow information and locate the attack host according to the switch ID and the switch ingress port in_port. If the attacking host exists, proceed to the next step. If the host does not exist, the neighbor controller is notified through the east–west interface of the controller, and the flow table information of the same source IP and destination IP field is searched in the edge switch, and the switch and switch port in_port that the attack host is connected to are located. The pseudocode of the algorithm is shown in Algorithm 3.
3. Flow rule generation. The flow rules are constructed with the matching field of in_port = the port to which the attack host is connected and the Action domain: Drop.
4. The flow table is issued. The flow rules constructed in step 3 are issued to the switch connected to the attacking host to achieve the isolation of the attacking host.

**Algorithm 3:** Attack source location algorithm**Input:** blacklist, network topology;**Output:** the edge switch port to which the attack source is connected(1) Extract_features (SrcIP, DstIP, switchID, in_port, t_stamp) from the blacklist (2) Results = Search (SrcIP & DstIP)(3) Infor1 = Sort_stamp (Results)(4) Infor2 = Extract_max_stamp (Infor1) (5) Extract switch_ID & in_port from infor2(6) **If** switch[i].inport is connected with a host (7) **then** the connected host is the attack host(8) **else** inform the other neighbor controllers about this flow and repeat step 2~7(9) **end If**


## 5. Experiment and Evaluation

### 5.1. Experimental Setup

**Figure 7:**
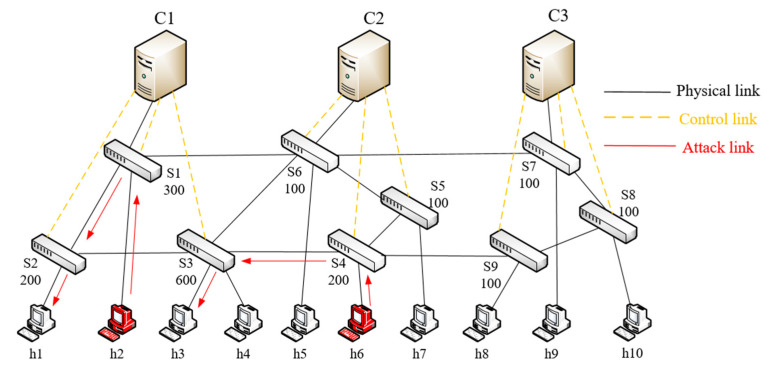
Network topology.

This experiment was conducted in the environment of Mininet [28], RYU [29] was selected as the controller, and CC-Guard was deployed in the controller. Its network topology is shown in Figure 7, where the controllers are C1 to C3, switches S1 to S9, and hosts h1 to h10. The hardware and OS are configured with an Intel Corei7-10510u CPU, 16 GB RAM, and Ubuntu 20.0.1. The CNN-GRU-Attention hybrid model is implemented through the Keras framework.

### 5.2. Performance Evaluation

#### 5.2.1. Load Migration Module

This experiment was simulated on Mininet, and the cbench [30] tool was used to measure the performance of each controller. The network topology is shown in Figure 7, with controllers C1 to C3, switches S1 to S9, and the number of packet_in messages processed in each switch. The cbench measurement results showed that the controller could process 1200 packet_in flow request packets per second (1200 pps) on average. The controller utilization threshold was set to 0.8, and a value greater than this threshold was considered the controller load.

**Figure 8:**
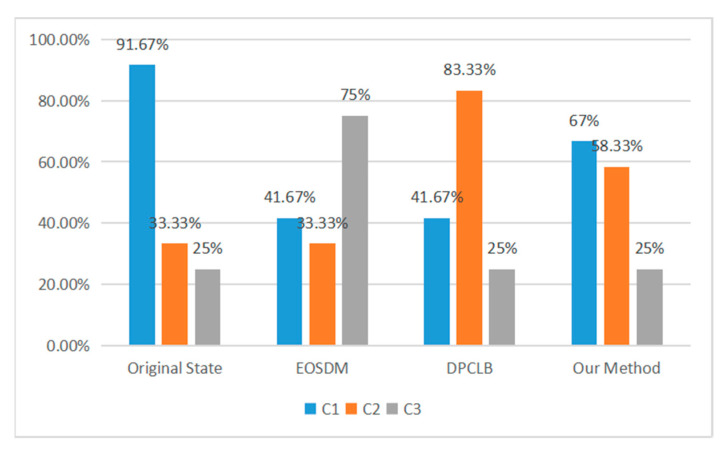
Controller load rate.

Through the switch migration strategy in this paper, we determined that the optimal migration scheme was as follows: migrating switch S1 under controller C1 to controller C2. To verify the superiority of the proposed switch migration strategy, it was compared with the EOSDM method [31] and DPCLB method [32]. The experimental results show that the proposed method achieved significant results in the controller load ratio (the ratio of the controller’s load to the controller’s maximum processing capacity), as shown in Figure 8.

#### 5.2.2. Anomaly Detection Module

In this paper, in order to effectively distinguish DDoS attacks in the SDN control layer, a two-stage attack detection method was used. In the first stage of detection, the experimental design was carried out according to Figure 7, and the attack target was controller C1. There were three switches and four hosts under the C1 controller. Among them, h2 and h6 were attack hosts that used the Hping3 tool to launch DDoS attacks on target hosts h1 and h3, respectively, and used the D-ITG tool to generate normal data flow and burst flow (flash flow). The abnormal flow was analyzed according to the description method in Section 4.4, and the experimental results are as follows.

In the first stage, the method of information entropy was used for coarse-grained detection. By calculating the entropy value of each feature vector in several windows, whether an anomaly occurred in the network was judged according to whether the entropy value exceeded the threshold. Firstly, the source IP address and the destination IP address of the packet_in messages collected under flash events and DDoS attacks comprised 50,000 data flows each. Then, the feature entropy values within each window (that is, 50 packets) were calculated by Equations (11) and (12). Finally, the confidence intervals of the entropy of source IP and destination IP were obtained by Equation (13), as shown in Table 4 and Table 5. According to the average confidence interval of the entropy of the source IP and the destination IP, the threshold of the source IP entropy could be determined as 2.5, and the threshold of the destination IP entropy was 1.0.

Through the above thresholds between the source IP entropy and the destination IP entropy, we could determine whether an anomaly occurred. If it were a flash event, the data flow would be forwarded normally. Otherwise, the system would adopt a deep learning approach for flow-based fine-grained detection.

In the second stage, the deep learning CNN-GRU-Attention method was used to detect DDoS attack flows. The model structure and parameters are shown in Figure 5 and Table 3. In this paper, we used the network topology in Figure 7 for sampling. We collected a total of 128,027 DDoS attack flows and 97,718 normal flows. Among them, 80% of the data was used as the training set and the remaining 20% of the data was used as the test set. To effectively evaluate the performance of the model, the 5-fold cross-validation method was used.

As different algorithms have different classification results, we compared different algorithms such as the deep neural network (DNN) classification algorithm, Adaboost classification algorithm, and CNN and GRU classification algorithms. The accuracy (*AC*), precision (*P*), recall (*R*), $F_{1}-\mathrm{Score}$ (*F*_1_) and false positive rate (*FPR*) of the different classifiers were also analyzed (where TP is true positive, TN is true negative, FP is false positive, and FN is false negative, and the experimental results are shown in Table 6.

- Accuracy rate (*AC*): indicates the number of samples correctly judged by the detection model as a percentage of the total number of input samples.
  (27) $$AC=\frac{TP+TN}{TP+TN+FP+FN}\times 100\%$$
- Precision (*P*): indicates the percentage of the number of samples that the detection model determines to be DDoS attack types that are actually DDoS attack types.
  (28) $$P=\frac{TP}{TP+FP}\times 100\%$$
- Recall (*R*): indicates the number of samples correctly determined by the detection model to be DDoS attack types as a percentage of the number of samples of all DDoS attack types.
  (29) $$R=\frac{TP}{TP+FN}\times 100\%$$
- F1-score (*F*_1_): represents the summed average of precision and recall, enabling a more accurate assessment of model performance.
  (30) F1=21p+1R×100%
- False positive rate (*FPR*): represents the percentage of the number of samples wrongly judged as the DDoS attack type to the total number of normal samples.
  (31) $$FPR=\frac{FP}{FP+TN}\times 100\%$$

The four detection metrics were similar for each classifier, so we used accuracy as an analysis example. The accuracy of CNN-GRU-Attention was 1.87%, 1.27%, 16.60%, 1.67%, and 0.45% higher than the other five common classifiers, respectively. The training time of CNN-GRU-Attention was relatively long, but the accuracy was improved by nearly 0.45–16.60%. Since the detection model was trained offline, it did not need to be updated frequently. It could accept a high training time while ensuring detection accuracy.

- AUC and ROC: To deal with imbalanced data, use the area under the curve (AUC) metric. AUC is the area of the receiver operating characteristic (ROC) curve. The ROC curve refers to a graph where the horizontal axis is the false positive rate and the vertical axis is the true positive rate. The maximum AUC value is 1, and the minimum AUC value is 0. The closer the AUC is to 1, the higher the accuracy; If its value is around 0.5, the prediction is not ideal enough.

**Figure 9:**
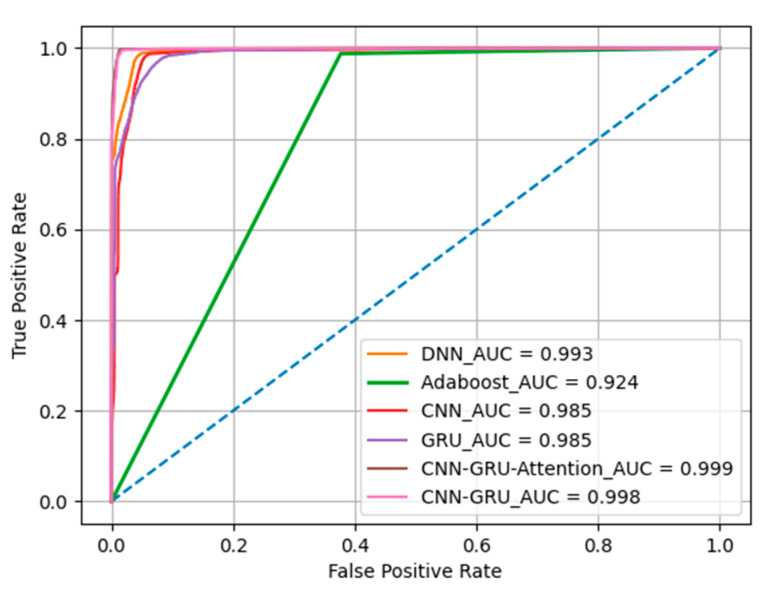
ROC curves for different detection algorithms.

Then, we compared the ROC with the AUC values for the five detection algorithms. Figure 9 shows that the ROC curve of CNN-GRU-Attention was steeper than that of DNN, Adaboost, CNN, and GRU, indicating that the CNN-GRU-Attention model performed better. The AUC value of DNN was 0.993, the AUC value of Adaboost was 0.924, the AUC values of CNN and GRU were 0.985, the AUC value of CNN-GRU was 0.998, and the AUC value of CNN-GRU-Attention was 0.999. The AUC value of CNN-GRU-Attention was higher than those of the other binary classification detection algorithm models, indicating that the proposed CNN-GRU-Attention anomaly detection algorithm has better performance.

- Confusion matrix: The confusion matrix is a metric to judge the results of the model and is used to judge the merits of the classifier. It is composed of two dimensions, namely “actual” and “prediction”, and both are composed of “true positive (TP)”, “true negative (TN)”, “false positive (FP)”, and “false negative (FN)”, as shown in Table 7.

**Figure 10:**
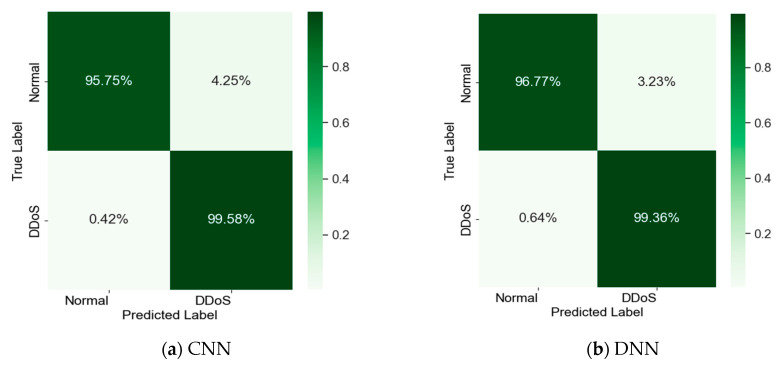

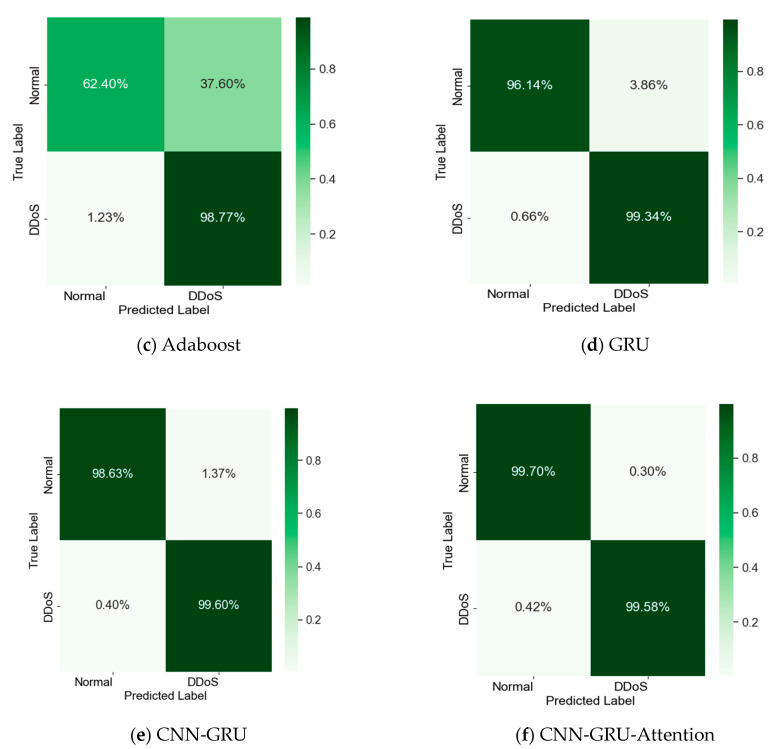
Comparison of confusion matrices: (**a**) CNN; (**b**) DNN; (**c**) Adaboost; (**d**) GRU; (**e**) CNN-GRU; (**f**) CNN-GRU-Attention.

The confusion matrices of the five classification algorithms are shown in Figure 10. CNN-GRU-Attention showed a DDoS attack false positive rate (FPR) of 0.30%, which was better than those of the other four detection methods. Since attacks can cause great harm to the network, the detection model requires high sensitivity to attacks. Therefore, CNN-GRU-Attention is more suitable as a binary classification anomaly detection algorithm than the other four detection algorithms.

#### 5.2.3. Mitigation Module

**Figure 11:**
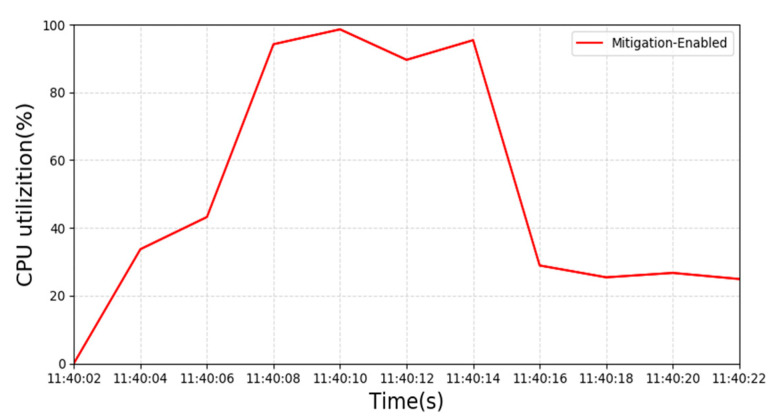
Trend diagram of controller CPU utilization over time.

Figure 11 shows that at approximately 11:40:06, malicious hosts h2, h3, and h5 launched DDoS attacks on h1. At this point, controller utilization started to increase until at 11:40:08, the utilization of the controller reached more than 90%. At approximately 11:40:14, CC-Guard started to take mitigation measures. At 11:40:16, the utilization of the controller returned to normal, indicating that the mitigation effect was successful.

## 6. Conclusions and Future Work

Aiming at the SDN architecture to avoid DDoS attacks, this paper proposes a security defense scheme, CC-Guard, with multi-domain controller cooperation. CC-Guard consists of four modules: the attack detection trigger module, the switch migration module, the detection module, and the mitigation module. The attack detection trigger module uses the controller’s ability to process packet_in messages as a prerequisite for triggering the CC-Guard system. This module can effectively reduce the time delay of the polling mechanism and execute the defense operation in time. In order to prevent controller congestion, the switch migration module can migrate the switches in the overloaded controller domain to the controller domain with idle resources, thus effectively avoiding controller overload and ensure the normal operation of the SDN network. The detection module uses a two-stage detection method. In the first stage, information entropy is used to distinguish flash events from DDoS attacks. Once an anomaly is suspected, the second phase of abnormal flow detection will be applied. In addition, to quickly detect attacks and detect them early, we use multiple IDS for parallel detection to improve detection efficiency. Finally, the mitigation module is started, the controller’s east–west interfaces are used for cross-domain traceability, and the OpenFlow protocol is used to issue flow rules to block the abnormal port of the switch. In order to effectively verify the defense effect of CC-Guard, this paper used Mininet and RYU controllers for simulation, and the experimental results verified the effectiveness of the CC-Guard defense system. In the DDoS attack environment, the system can not only accurately identify abnormal flow, but also effectively use the SDN controller for effective mitigation. Even if the SDN controller is congested, it can be guaranteed to work effectively.

As DDoS attacks become more and more intelligent, it is not foolproof to distinguish DDoS attacks and flash events by using the information entropy method in this paper. Therefore, it is urgent to propose a favorable solution that can accurately identify DDoS attacks and flash events. In addition, the network topology experiment in this article was small-scale and could not validate multiple IDS collaborative parallel detection schemes. In future work, we will conduct large-scale topology experiments to verify their effectiveness, and plan to deploy a physical SDN experimental platform for further research, attempting to improve the versatility and scalability of the CC-Guard framework.

## Figures and Tables

**Table 1:** List of key parameters in each module.

Parameter	Definition
$S_{i}$	The ith switch.
$C_{j}$	The jth controller.
$\lambda_{i}$	$\mathrm{The}\;\mathrm{flow}\;\mathrm{request}\;\mathrm{rate}\;\mathrm{of}\;\mathrm{switch}\;S_{i}$ in unit time t.
$\eta_{j}$	$\mathrm{The}\;\mathrm{processing}\;\mathrm{power}\;\mathrm{of}\;\mathrm{controller}\;C_{j}$ in unit time t.
δ	The controller starts the threshold of the detection module.
$\alpha_{i}$	$\mathrm{The}\;\mathrm{number}\;\mathrm{of}\;\mathrm{packets}\;\mathrm{in}\;\mathrm{messages}\;\mathrm{sent}\;\mathrm{by}\;\mathrm{switch}\;S_{i}$$\;\mathrm{to}\;\mathrm{controller}\;C_{j}$.
$f_{ij}$	$\mathrm{The}\;\mathrm{connection}\;\mathrm{relationship}\;\mathrm{between}\;\mathrm{switch}\;S_{i}\;\mathrm{and}\;\mathrm{controller}\;C_{j}$.
$H_{ij}$	$\mathrm{The}\;\mathrm{shortest}\;\mathrm{physical}\;\mathrm{distance}\;\mathrm{between}\;\mathrm{the}\;\mathrm{switch}\;S_{i}\;\mathrm{to}\;\mathrm{be}\;\mathrm{moved}\;\mathrm{and}\;\mathrm{the}\;\mathrm{overloaded}\;\mathrm{controller}\;C_{j}$.
$g_{ij}$	$\mathrm{The}\;\mathrm{connection}\;\mathrm{relationship}\;\mathrm{between}\;\mathrm{switch}\;S_{i}\;\mathrm{and}\;\mathrm{detection}\;\mathrm{system}\;IDS_{j}$.
$LD_{j}$	$\mathrm{The}\;\mathrm{load}\;\mathrm{of}\;\mathrm{the}\;\mathrm{detection}\;\mathrm{system}\;IDS_{j}$.
$\delta_{1}$	The threshold of the source IP entropy.
$\delta_{2}$	The threshold of the destination IP entropy.

**Table 2:** CNN-GRU-Attention model input features.

No	Name	Description	No	Name	Description
1	In_port	Ingress port	13	Cookie	Cookie
2	Eth_type	Ethernet type	14	priority	Flow rule priority
3	Eth_src	Ethernet source address	15	idle_timeout	Idle timeout
4	Eth_dst	Ethernet destination address	16	hard_timeout	Hard timeout
5	IP protocol	IP protocol	17	n_packets	Number of packets
6	IP ToS bits	IP ToS bits	18	n_bytes	Number of bytes
7	Src_ip	Source IP address	19	actions	Action of switch
8	Dst_ip	Destination IP address	20	avg_packets	Average of the number of packets
9	tcp_src/udp_src	TCP/UDP source port	21	avg_bytes	Average of packet sizes
10	tcp_dst/udp_dst	TCP/UDP destination port	22	avg_duration	Average duration of the flow
11	VLAN id	VLAN id	23	survival_degree	Survival of the flow
12	VLAN priority	VLAN priority	24	ration_packets	Ration of packets up and down

**Table 3:** CNN-GRU-Attention network architecture parameters.

Layer	Parameter
InputLayer	Input_shape = (24.1)
Conv1D + RELU	filters = 64, kernel = 3
MaxPooling1D	pool_size = 2
Conv1D + RELU	filters = 128, kernel = 3
MaxPooling1D	pool_size = 2
GRU	units = 64, return_sequences = True
Dropout	Rate = 0.5
AttentionLayer	none
Flatten	none
Dense + Softmax	units = 2

**Table 4:** Confidence interval of source IP entropy.

Entropy of the Source IP	Abnormal Flow	Flash Flow
Mean value	3.9098	2.5140
Standard deviation	0.3908	0.5208
Maximum value of confidence interval	3.934	2.5462
Minimum value of confidence interval	3.8856	2.4818
Threshold	2.5	

**Table 5:** Confidence interval of destination IP entropy.

Entropy of the Destination IP	Abnormal Flow	Flash Flow
Mean value	2.4957	0.9675
Standard deviation	1.1622	0.2689
Maximum value of confidence interval	2.5677	0.98416
Minimum value of confidence interval	2.4237	0.9508
Threshold	1.0	

**Table 6:** CNN-GRU-Attention model comparison results.

Model	Accuracy	Precision	Recall	F1-Score	FPR	Training Time
CNN	97.76%	97.01%	99.58%	98.27%	4.25%	264.51 s
DNN	98.36%	97.50%	99.36%	98.42%	3.23%	83.68 s
Adaboost	83.03%	77.49%	98.77%	86.85%	37.60%	4.27 s
GRU	97.96%	97.14%	99.34%	98.23%	3.86%	90.34 s
CNN-GRU	99.18%	98.97%	99.60%	99.28%	1.37%	347.12 s
CNN-GRU-Attention	99.63%	99.77%	99.58%	99.67%	0.30%	335.26 s

**Table 7:** Table of confusion matrix structure.

Confusion Matrix		Predicted Class	
		Negative	Positive
TrueClass	Negative	TN	FP
	Positive	FN	TP

## Data Availability

The datasets generated and analyzed during the current study are available from the corresponding author on reasonable request.

## References

[1] Nguyen V., Brunstrom A., Grinnemo K. (2017). SDN/NFV-Based Mobile Packet Core Network Architectures: A Survey. IEEE Commun. Surv. Tutor..

[2] Varadharajan V., Karmakar K., Tupakula U. (2019). A Policy-Based Security Architecture for Software Defined Networks. IEEE Trans. Inf. Forensics Secur..

[3] Bera P., Saha A., Setua S. Denial of Service Attack in Software Defined Network. Proceedings of the 5th International Conference on Computer Science and Network Technology (ICSNT).

[4] OpenFlow Switch Specifification V1.4.0. https://www.opennetworking.org/wp-content/uploads/2014/10/openflow-spec-v1.4.0.pdf.

[5] Kiranyaz S., Avci O., Abdeljaber O. (2021). 1D convolutional neural networks and applications: A survey. Mech. Syst. Signal Process..

[6] Assis M., Carvalho L., Loret J. (2021). A GRU deep learning system against attacks in software defined networks. J. Netw. Comput. Appl..

[7] Cao Y., Deng Y., Wu J. (2022). Detdcting and Mitigating DDoS Attacks in SDN Using Spatial-Temporal Graph Convolutional Network. IEEE Trans. Dependable Secur. Comput..

[8] Agha S., Rehman O. Improving Discrimination Accuracy Rate of DDoS Attacks and Flash Events. Proceedings of the 2020 International Conference on Cyber Warfare and Security (ICCWS).

[9] Nam T., Phong P., Khoa T. Self-organizing map-based approaches in DDoS flooding detection using SDN. Proceedings of the 32nd International Conference on Information Networking (ICOIN).

[10] Deepa V., Sudar K., Deepalakshmi P. Design of Ensemble Learning Methods for DDoS Detection in SDN Environment. Proceedings of the 2019 International Conference on Vision Towards Emerging Trends in Communication and Networking (Vi-TECoN).

[11] Li C., Wu Y., Qian Z. (2018). DDoS attack detection and defense based on hybrid deep learning model in SDN. J. Commun..

[12] Cui Y., Yan L., Li S. (2016). SD-Anti-DDoS: Fast and efficient DDoS defense in software-defined networks. J. Netw. Comput. Appl..

[13] Yang X., Han B., Sun Z. SDN-Based DDoS Attack Detection with Cross-Plane Collaboration and Lightweight Flow Monitoring. Proceedings of the IEEE Global Telecommunications Conference (Globecom).

[14] Cui J., He J.T., Xu Y. TDDAD: Time-based detection and defense scheme against DDoS attack on SDN controller. Proceedings of the 23rd Australasian Conference on Information Security and Privacy (ACISP).

[15] Zhang L., Wang J. (2022). A hybrid method of entropy and SSAE-SVM based DDoS detection and mitigation mechanism in SDN. Comput. Secur..

[16] Shin S., Yegneswaran V., Porras P. AVANT-GUARD: Scalable and vigilant switch flow management in software-defined networks. Proceedings of the2013 ACM SIGSAC Conference on Computer and Communication Security.

[17] Wang H., Lei X., Gu G. FloodGuard: A DoS Attack Prevention Extension in Software-Defined Networks. Proceedings of the45th Annual IEEE/IFIP International Conference on Dependable Systems & Networks.

[18] Gao S., Peng Z., Xiao B. (2020). Detection and Mitigation of DoS Attacks in Software Defined Networks. IEEE/ACM Trans. Netw..

[19] Macedo R., Castro R., Santos A. Self-Organized SDN Controller Cluster Conformations Against DDoS Attacks Effects. Proceedings of the 2016 IEEE Global Communications Conference (GLOBECOM).

[20] Wang Y., Hu T., Tang G. (2019). SGS: Safe-Guard Scheme for Protecting Control Plane Against DDoS Attacks in Software-Defined Networking. IEEE Access.

[21] Wu P., Yao L., Lin C., Wu G., Obaidat M.S. (2018). FMD: A DoS mitigation scheme based on flow migration in software-defined networking. Int. J. Commun. Syst..

[22] Jiang S., Yang L., Gao X. (2022). BSD-Guard: A Collaborative Blockchain-Based Approach for Detection and Mitigation of SDN-Targeted DDoS Attacks. Secur. Commun. Netw..

[23] Shalini P., Radha V., Sanjeevi S. (2022). DOCUS-DDoS detection in SDN using modified CUSUM with flash traffic discrimination and mitigation. Comput. Netw..

[24] Wang F., Xu G., Wang M. (2023). An Improved Genetic Algorithm for Constrained Optimization Problems. IEEE Access.

[25] Liu Y., Zhi T., Shen M. (2022). Software-defined DDoS detection with information entropy analysis and optimized deep learning. Future Gener. Comput. Syst..

[26] Elsayed M.S., Le-Khac N.-A., Albahar M. (2021). A novel hybrid model for intrusion detection systems in SDNs based on CNN and a new regularization technique. J. Netw. Comput. Appl..

[27] Laghrissi F., Douzi S., Douzi K. (2021). IDS-attention: An efficient algorithm for intrusion detection systems using attention mechanism. J. Big Data.

[28] Erel M., Teoman E., Ozcevik Y. Scalability analysis and flow admission control in mininet-based SDN environment. Proceedings of the2015 IEEE Conference on Network Function Virtualization and Software-Defined Networks (NFV-SDN).

[29] Kubo R., Fujita T., Agawa Y. (2014). Ryu SDN framework-open-source SDN platform software. NTT Tech. Rev..

[30] Jawaharan R., Mohan P., Das T. Empirical Evaluation of SDN Controllers Using Mininet/Wireshark and Comparison with Cbench. Proceedings of the 27th International Conference on Computer Communication and Network (ICCCN).

[31] Yao L., Hu T., Peng Y. (2019). Switch Dynamic Migration Strategy Based on Efficiency Optimization in SDN. Acta Electron. Sinica.

[32] Hu T., Zhang J., Wu J. (2018). Controller Load Balancing Mechanism Based on Distributed Policy in SDN. Acta Electron. Sinica.

